# Awareness of colorectal cancer and screening uptake in Eastern KSA: Predictors and implications

**DOI:** 10.1016/j.jtumed.2026.02.001

**Published:** 2026-02-25

**Authors:** Saleh A. Busbait, Shadi A. Alshammary, Badour A. Alzahrani, Masoma A. Al-houri, Loay M. Bojbara, Humood A. Alsadery, Othman Y. AlFrayyan, Hassan A. Alsaleem

**Affiliations:** Department of Surgery, College of Medicine, Imam Abdulrahman Bin Faisal University, Dammam, KSA

**Keywords:** Cancer screening, Colorectal cancer, Colorectal cancer awareness, Screening uptake, فحص السرطان, سرطان القولون والمستقيم, الوعي بسرطان القولون والمستقيم, الاقبال على الفحص المبكر

## Abstract

**Objectives:**

Colorectal cancer (CRC) is one of the most frequent cancers in KSA, ranking first among males and third among females. However, the uptake of the national screening programs is poor due to insufficient awareness and access barriers. Thus, this study evaluated the awareness of CRC and screening levels among adults in the Eastern Province of KSA, where recent data are limited.

**Methods:**

A cross-sectional study was conducted among 412 adults using a self-administered questionnaire between April 2024 and July 2024. Awareness of CRC signs, symptoms, risk factors, and screening was assessed. Logistic regression was used to determine the predictors of awareness and screening practice.

**Results:**

In total, 57.0 % of the participants had low awareness of CRC. Females were more aware of the signs and symptoms of CRC than males (*p* = 0.025). The lowest age group (18–30 years) had the highest mean awareness score (*p* = 0.037). Higher level of education was positively related to knowledge of risk factors for CRC (*p* < 0.001). Only 11.7 % had undergone CRC screening, which increased to 24.7 % among those aged 50 years and above. Positive predictors of awareness included higher education level (*p* = 0.023) and prior knowledge of CRC (*p* = 0.043). Age (*p* < 0.001), living in main (central urban areas) areas (*p* < 0.001), and family history (*p* < 0.001) were predictors of undergoing screening.

**Conclusions:**

In the Eastern Province of KSA, awareness of CRC and screening rates are low. Thus, it is suggested that awareness campaigns, discussions with physicians, and electronic medical records-based reminder systems may help enhance compliance.

## الملخص

**المقدمة:**

يُعد سرطان القولون والمستقيم من أكثر أنواع السرطان شيوعًا في المملكة العربية السعودية، حيث يحتل المرتبة الأولى بين الذكور والثالثة بين الإناث. وعلى الرغم من وجود برامج وطنية للفحص المبكر، إلا أن معدلات الوعي والمشاركة في الفحص لا تزال منخفضة، مما يعكس الحاجة لفهم مستوى المعرفة الحالي وتحديد العوامل المؤثرة على السلوك الصحي المتعلق بالفحص.

**الأهداف:**

هدفت هذه الدراسة إلى تقييم مستوى الوعي بسرطان القولون والمستقيم، بما في ذلك الأعراض وعوامل الخطورة، وكذلك مدى انتشار فحص الكشف المبكر، وتحليل العوامل الديموغرافية المرتبطة بهما بين البالغين في المنطقة الشرقية من المملكة.

**المنهجية:**

تم إجراء دراسة مقطعية شملت ٤١٢ مشاركًا من البالغين باستخدام استبيان إلكتروني موزع بين أبريل ويوليو ٢٠٢٤. تم تقييم الوعي بالأعراض وعوامل الخطورة والفحص، وتحليل العوامل المتنبئة بها. شملت البيانات الخصائص الديموغرافية، مستوى التعليم، وجود تاريخ عائلي للسرطان، والمكان الجغرافي.

**النتائج:**

أظهرت النتائج أن ٥٧٪ من المشاركين لديهم وعي ضعيف بسرطان القولون والمستقيم. كانت الإناث أكثر وعيًا بالأعراض مقارنةً بالذكور، وسجلت الفئة العمرية من ١٨ إلى ٣٠ عامًا أعلى متوسط للوعي. وُجد ارتباط إيجابي بين التعليم العالي والوعي بعوامل الخطورة. بلغت نسبة من خضعوا للفحص المبكر للقولون ١١.٧٪ فقط، وارتفعت إلى ٢٤.٧٪ بين من تجاوزوا ٥٠ عامًا. من العوامل المتنبئة بالوعي الجيد: التعليم العالي، والتعرض المسبق لمعلومات حول السرطان، ووجود تاريخ عائلي. بينما كان العمر المتقدم، والإقامة في مناطق حضرية، ووجود تاريخ عائلي عوامل متنبئة بزيادة احتمالية الخضوع للفحص.

**الاستنتاج:**

تعكس الدراسة تدني مستويات الوعي والمشاركة في فحص سرطان القولون والمستقيم في المنطقة الشرقية، مما يستدعي تدخلات متعددة المستويات تشمل حملات توعوية موجهة، تعزيز دور الأطباء في التوصية بالفحص، وتفعيل أنظمة تذكير إلكترونية ضمن السجلات الطبية. تمثل هذه الخطوات ركائز لتعزيز الالتزام المجتمعي ببرامج الفحص وتقليل عبء المرض في المملكة.

## Introduction

Colorectal cancer (CRC) is one of the most common malignancies worldwide. In KSA, CRC ranks first in males accounting for 17.2 % of all cancers and third in females accounting for 9.2 %.^1^ Survival and the prognosis is highly correlated with the stage of presentation for CRC. The incidence of CRC has been increasing over time. Early stage CRC and polyps tend to be asymptomatic, thereby highlighting the important role of screening programs in KSA and increasing awareness.^1^^,^^2^ A previous study demonstrated a positive association between an individual's level of knowledge about CRC and their participation in screening programs.^3^ Adherence to screening can be influenced by the patient, healthcare system, provider, and policy level factors.^1^

A recent Saudi cohort by Alreshidi et al. linked the increasing incidence of CRC to lifestyle risk factors, such as obesity and dietary habits.^4^ Alharbi et al. reported that studies from KSA and neighboring Gulf countries consistently found an increasing incidence of CRC and limited awareness of screening modalities despite national screening programs.^5^

The Saudi Ministry of Health launched a national CRC screening program that recommends an annual fecal immunochemical test (FIT) for adults aged 45–75 years, followed by colonoscopy for those with positive results.^6^ This initiative aligns with the Health Sector Transformation Program (2021–2025) under Vision 2030, which emphasizes preventive care, early detection, and improved access to screening services.^6^ Most previous Saudi studies that assessed awareness of CRC were conducted in the Central and Western regions, and the Eastern Province was underrepresented. Thus, in the present study, we addressed this deficiency to provide evidence to guide locally tailored public health interventions. The aim of this study was to evaluate the awareness of CRC and screening levels among the community in the Eastern Province of KSA, particularly regarding the incidence of CRC, risk factors, symptoms, screening, and management relative to different demographic data. The findings obtained in this study will assist with the development of appropriate educational interventions to increase the uptake of CRC screening programs.

## Materials and Methods

This cross-sectional study was conducted to evaluate the awareness of CRC and screening levels of colorectal cancer (CRC) among inhabitants of the Eastern Province Region of KSA. We used a self-administered electronic survey, which was distributed through social media and in public places, mainly shopping malls. The study targeted individuals who satisfied the following eligibility criteria: living in the Eastern Province of KSA, not diagnosed with CRC or inflammatory bowel disease, and aged at least 18 years. Individuals who did not satisfy the eligibility criteria or were illiterate were excluded. The required sample size was estimated as 385 participants using the Raosoft calculator, with a 5 % margin of error and 95 % confidence interval (CI). Data were collected between April 2024 and July 2024.

The questionnaire was based on established tools provided by the American Cancer Society and previous studies conducted in the area by Galal et al.^2^^,^^7^ The survey consisted of three main parts: sociodemographic data, CRC related knowledge and behaviors, and knowledge of the signs, symptoms, and risk factors of CRC. The sociodemographic section collected data regarding age, sex, area of residence, and educational level. Residence status was further distinguished as core (main) for central urban areas and peripheral areas based on the provincial administrative divisions of KSA. In this study, CRC-related behaviors refer primarily to the screening practices of participants and family-related risk awareness. The survey included questions about CRC awareness, family history of CRC, screening experiences, screening timing, and screening techniques (endoscopy, fecal occult blood test, and computed tomography (CT)). The CRC related knowledge section assessed the knowledge of participants regarding CRC, family history of CRC, screening history, screening methods, and age at screening. CRC symptoms and risk factors were measured using six items for symptoms and 10 items for risk factors. Awareness was measured directly using a scoring system derived from 16 items (six for symptoms and 10 for risk factors). Awareness classification was based on the total scores, where those with a score ≥9 points (out of 16) were categorized as having “Good” awareness and those with a score <9 as having “Poor” awareness. In this study, awareness was considered to refer to knowledge-based awareness, including understanding of CRC symptoms and risk factors, rather than simple recognition of the disease's name. This classification method was employed in previous studies of CRC awareness.^2^ The questionnaire was pretested with 14 randomly selected individuals to ensure its clarity and internal consistency. The Cronbach's alpha score of 0.74 demonstrated the satisfactory internal consistency of the instrument. The questionnaire was written in English, translated into Arabic by two independent experts, and back-translated into English by a third expert to verify its accuracy and clarity.

IBM SPSS version 30.0 was used for data analysis. Descriptive statistics were used to summarize participants’ characteristics, awareness levels, and screening behaviors. Chi-square tests and non-parametric tests (Mann–Whitney U and Kruskal–Wallis tests) were used to compare awareness and screening uptake across demographic groups. Logistic regression analysis was conducted to distinguish the independent predictors of CRC awareness and screening behavior, with adjustment for potential confounders. The study involved no physical, psychological, social, legal, or economic risks to participants. Approval for the study was obtained from the Institutional Review Board (IRB) before data collection commenced. Participants were informed about the study and consented voluntarily, with anonymity guaranteed.

### Abbreviations

CRC, Colorectal Cancer; FIT, Fecal Immunochemical Test.

## Results

In total, 412 participants were included in the study, where the majority were male (70.6 %) and resided in core (main) areas (93.9 %) (Table 1). The mean awareness score for CRC signs and symptoms was 3.71 ± 1.34 (out of 6) and the mean risk factors score was 4.38 ± 2.20 (out of 10). Awareness levels varied significantly across different demographic groups (Table 2).

**Table 1:** Sociodemographic characteristics and colorectal cancer (CRC) screening uptake.

Sociodemographic and CRC screening variables	Number	%
**Age**		
18–30	62	15.0
31–40	101	24.5
41–50	108	26.2
51–60	88	21.4
>60	53	12.9
**Total**	**412**	**100**
**Sex**		
Male	291	70.6
Female	121	29.4
**Total**	**412**	**100**
**Residence**		
Core (main)	387	93.9
Peripheral	25	6.1
**Total**	**412**	**100**
**Education level**		
Primary	3	0.7
Intermediate	4	1.0
Secondary	73	17.7
University	272	66.0
Higher education	60	14.6
**Ever heard of CRC?**	370	89.8
**Family history of CRC?**	86	20.9
**Ever screened for CRC?**	48	11.7
**Good awareness (score ≥ 9)**	177	43.0

**Table 2:** Awarenes^a^s of colorectal cancer (CRC) signs/symptoms and risk factors by participant characteristics.

Variable	Mean signs/symptoms score (SD)	*p*-value	Mean risk factors score (SD)	*p*-value
**Sex**				
Male	3.48 ± 1.37	0.025	4.37 ± 2.32	0.671
Female	3.81 ± 1.31		4.47 ± 2.06	
**Age group**				
18–30	3.80 ± 1.42	0.037∗	4.89 ± 2.13	0.083∗
31–40	3.75 ± 1.29		4.68 ± 2.01	
41–50	3.41 ± 1.39		4.30 ± 2.08	
51–60	3.60 ± 1.36		4.11 ± 2.36	
>60	3.35 ± 1.28		3.96 ± 2.11	
**Education**				
Primary school	3.21 ± 1.42	0.253	3.35 ± 2.25	<0.001∗
Intermediate	3.50 ± 1.39		4.00 ± 2.30	
Secondary	3.55 ± 1.32		4.16 ± 2.15	
University	3.72 ± 1.31		4.91 ± 2.08	
Higher education	3.85 ± 1.28		5.30 ± 2.10	
**Residence**				
Core (main)	3.56 ± 1.36	0.193	4.41 ± 2.25	0.649
Peripheral	3.92 ± 1.26		4.20 ± 2.14	
**Family history of CRC**				
Yes	3.79 ± 1.38	0.817	4.59 ± 2.11	0.573
No	3.75 ± 1.32		4.40 ± 2.22	
**Ever heard of CRC?**				
Yes	3.79 ± 1.33	0.041∗	4.55 ± 2.19	0.044∗
No	3.43 ± 1.40		4.01 ± 2.16	
**Ever screened for CRC?**				
Yes	3.89 ± 1.38	0.324	4.67 ± 2.15	0.395
No	3.72 ± 1.34		4.38 ± 2.20	

a ∗ Statistically significant at p< 0.05

Females had a higher mean awareness score for signs and symptoms (3.81 ± 1.31) compared with males (3.48 ± 1.37, *p* = 0.025), but no significant difference was observed in risk factor awareness between sexes (*p* = 0.671). Younger participants (aged 18–30 years) had the highest mean awareness scores for signs and symptoms (3.80 ± 1.42, *p* = 0.037), and the scores tended to decease in older age groups. A similar but non-significant pattern was found for risk factor awareness (*p* = 0.083). Higher education was significantly associated with greater risk factor awareness (*p* < 0.001), but the association with signs and symptoms awareness was not significant (*p* = 0.23). Having a family history of CRC was not significantly associated with awareness of signs and symptoms (*p* = 0.817) or risk factors (*p* = 0.573). Moreover, participants who had heard about CRC before the survey had significantly higher awareness scores for both signs and symptoms (*p* = 0.041) and risk factors (*p* = 0.044) (Table 2).

Overall, only 11.7 % of participants had undergone CRC screening, where the uptake of screening increased to 24.7 % among those aged 50 years and older (Table 1). The CRC screening methods used for participants varied, where colonoscopy was the most common screening modality (77.1 %), followed by FIT (41.7 %), CT (12.5 %), and other methods (8.3 %). The majority (56.3 %) were screened between the ages of 50 and 60 years (Table 3).

**Table 3:** Colorectal cancer (CRC) screening methods and age at screening.

Screening variable	Number	%
**Age at screening**		
<40 years	4	8.3
40–50 years	13	27.1
50–60 years	27	56.3
≥60 years	4	8.3
**Total**	**48**	**100**
**Screening method used**		
Colonoscopy	37	77.1
FIT	20	41.7
CT	6	12.5
Other	4	8.3
**Total**	**48**	**100**
**Multiple methods used**	15	31.2

Logistic regression analysis identified significant predictors of good CRC awareness and screening behavior, which explained 10.8 % of the variance (Nagelkerke R^2^ = 0.108) and the model accuracy was 63.8 %. Higher education (*p* = 0.023), having a family history of CRC (*p* = 0.006), and prior knowledge of CRC (*p* = 0.043) were significantly associated with better awareness. However, sex and residence area were not identified as significant predictors (Table 4).

**Table 4:** Predictors of good colorectal cancer (CRC) awareness (logistic regression model, awareness category as outcome).

Variable^a^	Odds ratio (95 % CI)	*p*-value
Sex	1.033 (0.663–1.611)	0.892
**Age group**		
18–30 (ref)	1.00	–
31–40	0.982 (0.476–2.027)	0.961
41–50	0.433 (0.221–0.849)	0.016∗
51–60	0.458 (0.224–0.940)	0.035∗
>60	0.661 (0.290–1.509)	0.327
**Education level**		0.023∗
Primary (ref)	1.00	–
Intermediate	1.211 (0.207–7.087)	0.827
Secondary	1.705 (0.328–8.874)	0.525
University	2.498 (0.477–13.067)	0.277
Higher education	4.067 (0.705–23.466)	0.112
**Residence**		0.731
Peripheral (ref)	1.00	–
Core (main)	1.156 (0.522–2.561)	0.731
**Family history of CRC**	3.037 (1.374–6.717)	0.006∗
**Ever heard of CRC?**	2.050 (1.022–4.108)	0.043∗
**Ever screened for CRC?**	1.000 (0.435–2.298)	0.999

a ∗ Statistically significant at p <0.05

Age was a strong predictor of screening uptake, where participants aged 51–60 years (odds ratio (OR): 12.72, *p* = 0.016) and those above 60 years (OR: 18.35, *p* = 0.007) were significantly more likely to have undergone screening compared with those in younger groups. Education level was not a significant predictor of screening uptake (*p* = 0.366), suggesting that factors other than education, such as access and health system barriers, may have been more important. The likelihood of being screened was significantly higher for participants who resided in core areas (OR: 4.92, 95 % CI: 2.281–10.615, *p* < 0.001) and those with a family history of CRC (OR: 4.92, 95 % CI: 2.268–10.695, *p* < 0.001). The screening model explained 29.8 % of the variance (Nagelkerke R^2^ = 0.298) and the overall accuracy was 87.6 % (Table 5).

**Table 5:** Predictors of colorectal cancer (CRC) screening uptake (logistic regression model, screening status as outcome).

Variable	Odds ratio (95 % CI)	*p*-value
**Sex**	2.39 (0.946–6.041)	0.065
**Age group**		<0.001∗
18–30 (ref)	1.00	–
31–40	1.432 (0.152–13.505)	0.764
41–50	4.46 (0.527–37.742)	0.174
51–60	12.72 (1.576–102.6)	0.016∗
>60	18.35 (2.292–146.9)	0.007∗
**Education level**		0.366
Primary (ref)	1.00	–
Intermediate	0.176 (0.012–2.693)	0.215
Secondary	0.134 (0.010–1.762)	0.123
University	0.243 (0.015–3.966)	0.309
Higher education	0.823 (0.179–3.793)	0.804
**Residence**		<0.001∗
Peripheral (ref)	1.00	–
Core^a^	4.92 (2.281–10.615)	<0.001∗
**Family history of CRC**	4.92 (2.268–10.695)	<0.001∗

a ∗ Statistically significant at p <0.05

## Discussion

This study aimed to explore levels of awareness of the signs, symptoms, and risk factors of CRC, as well as screening uptake and its predictors among adults. The findings showed that the levels of awareness and rates of screening were low, with key differences according to demographics and prior exposure to CRC related information.

Our findings showed that CRC awareness was low, where 57.0 % of participants had poor awareness. Similarly, previous studies in KSA and other Gulf countries found limited knowledge of the signs, symptoms, and risk factors of CRC.^2^^,^^3^^,^^8^, ^9^, ^10^, ^11^ It was notable that females had significantly higher awareness of the signs and symptoms of CRC compared with males (*p* = 0.025). However, risk factor awareness did not differ between sexes, and thus disparities in general health knowledge did not necessarily translate into risk perception.

A worrying trend was the decreasing awareness scores among the older participants, where awareness levels were highest for younger individuals (18–30 years). This pattern indicates that younger generations could have benefited from more contact with digital health resources and social media awareness campaigns for CRC.^11^ However, if older adults with a higher risk of CRC lack the same access to or engagement with these educational materials, then there is a clear need for specific awareness efforts targeting older populations.

We included participants aged under 45 years in this study because we wanted to understand how this age group perceived CRC screening. Younger adults were included to understand their awareness patterns as early-onset CRC cases continue to rise. Recent global analyses have documented rising early-onset CRC rates in those aged below 50 years throughout Asia and the Middle East, and thus educational programs and preventive measures are required immediately before screening becomes necessary.^12^

Education was a strong predictor of CRC awareness, especially regarding risk factors (*p* < 0.001). Increasing education level tended to be associated with better awareness of signs and symptoms of CRC, but this relationship was not statistically significant. Therefore, formal education is a determinant of general health literacy but it might not result in comprehensive knowledge of the symptoms of a particular disease unless reinforced by targeted health campaigns and direct exposure to health information.^10^^,^^13^

Logistic regression further highlighted education and prior exposure to CRC-related information as key predictors of overall awareness. Participants with higher education (*p* = 0.023) were more likely to have good awareness than those with lower education, which supports the idea that formal education is a key factor for determining health literacy, especially regarding risk factors. Furthermore, those with a family history of CRC (*p* = 0.006) exhibited greater awareness, probably due to personal or familial experience making the disease more noticeable. Prior knowledge of CRC (*p* = 0.043) was also found to be a good predictor, thereby indicating that contact with information in the media, healthcare professionals, or public campaigns can increase awareness of the disease. Interestingly, sex and residence were not significant predictors, demonstrating that lack of awareness persists in both urban and rural areas, as well as in men and women. Thus, education is a factor in awareness but other structural and cultural factors are also important, so targeted interventions are required that are not based only on formal education.

Despite increased awareness in some groups, CRC screening uptake was alarmingly low, where only 11.7 % of participants reported prior screening. Among those aged 50 years and older, 24.7 % had undergone CRC screening, and although this was higher than the overall rate, there are clearly still issues with adherence to the recommended screening guidelines.^2^^,^^14^^,^^15^ Logistic regression analysis identified age, residence, and family history of CRC as key predictors of screening uptake. Individuals aged 51–60 years (OR: 12.72, *p* = 0.016) and those aged >60 years (OR: 18.35, *p* = 0.007) were significantly more likely to have undergone screening, aligning with age-based screening recommendations. The odds of screening were nearly five times higher among participants who resided in main areas (OR: 4.92, *p* < 0.001), possibly reflecting the better healthcare access in urban settings. A family history of CRC was also a strong predictor of screening (OR: 4.92, *p* < 0.001), emphasizing the role of perceived personal risk in driving screening behaviors. However, education level was not significantly associated with screening uptake, suggesting that barriers other than knowledge, such as accessibility, affordability, and cultural factors, may have more critical roles.

Our findings agree with those obtained in previous studies conducted in KSA and the Gulf region, highlighting the suboptimal awareness of CRC and screening rates, and reinforcing the need for targeted awareness campaigns.^2^^,^^3^^,^^8^, ^9^, ^10^, ^11^ A study in the Southern region of KSA found that 61 % of the participants were aware of the symptoms of CRC, but only 45 % could describe the risk factors, which are similar to our findings (43.0 % had good awareness).^16^ Moreover, a cross-sectional study in the Western region of KSA found that the awareness rate for CRC was 36.6 %.^15^ Another study conducted in the Eastern Province of KSA among older adults showed that 33.6 % of the participants were aware of CRC.^2^ In our sample, we also found that younger people, especially those aged 18–30 years, had greater awareness scores than older participants, as also observed in studies conducted in KSA and the Gulf region.^8^, ^9^, ^10^, ^11^^,^^13^, ^14^, ^15^, ^16^ This trend indicates that the younger generation may have easier access to health information and campaigns on the Internet than the older generation who may need specific educational interventions.^17^

We found a positive correlation between the education level and knowledge of CRC, especially regarding risk factors (*p* < 0.001). A similar study of Saudi medical students by Althobaiti et al. and a population-based study by Elmaghraby et al. both showed that increasing levels of education were associated with better knowledge of CRC.^10^^,^^11^^,^^13^ This supports the need to include CRC education in academic curricula and public health campaigns to improve awareness.^18^ Our results showed that the CRC screening rate was 11.7 %, rising to 24.7 % among participants aged 50 years and older. These results are consistent with previous studies in the area, where one conducted in Eastern Province found that the CRC screening rates were generally low.^8^^,^^14^ The screening rates were also reported to be less than 20 % in various studies conducted in the Qassim and Al-Baha regions, although the national guidelines recommend screening for early detection.^16^^,^^19^

The Saudi Ministry of Health has established a national CRC screening program by using the FIT to identify occult blood in stool samples. This non-invasive annual test is recommended for people aged 45–75 years, and a positive FIT result requires referral for colonoscopy to exclude malignancy.^20^ However, the effectiveness of this program is hindered by low participation rates due to challenges including supply–demand mismatches, manpower and facility limitations, and inadequate patient follow-up.^10^^,^^20^^,^^21^

Addressing these operational challenges is crucial for enhancing participation rates and improving the program's overall effectiveness in reducing the incidence of CRC and mortality in KSA. Predictors of screening uptake identified in our study include: older age (51–60 years: OR = 12.72, *P* = 0.016; >60 years: OR = 18.35, *p* = 0.007) and family history of CRC (OR = 4.92, *p* < 0.001). Similar predictors have been identified in studies across the Gulf region, indicating that screening behaviors are affected by direct or indirect contact with CRC through personal or familial experience.^2^^,^^13^

However, our study did not establish a clear relationship between sex and screening uptake, despite the contradictory findings obtained in previous studies. For instance, Galal et al. argued that women were less likely to undergo screening because of fear, embarrassment, and discomfort with the procedure.^2^ In addition, Khayyat et al. highlighted these factors, as well as anxiety about the results of the tests, as major determinants of non-participation in screening programs by women.^22^

In particular, the strong association between prior awareness of CRC and higher screening rates in our study (*p* = 0.043) highlights the importance of public health campaigns targeting CRC awareness. Studies by Alsaigh et al. and Al Abdouli et al. also demonstrated that people with better knowledge of CRC were more likely to undergo screening than those with limited knowledge.^11^^,^^19^

Thus, our findings can be considered to add to the growing evidence regarding the effectiveness of targeted interventions aimed at enhancing awareness of CRC and screening compliance in KSA. Moreover, future studies should focus on how to align people's knowledge and their actions, so rising awareness will lead to higher rates of screening.

Increasing awareness could have a major impact on increasing early detection rates if awareness programs are integrated into schools, universities, and workplaces. Public health strategies to promote CRC education have been shown to increase participation in screening.^23^ From a policy perspective, incorporating CRC screening reminders into electronic medical records may also help to improve participation rates, as recommended by national guidelines.^20^ The increasingly older population will lead to a rise in CRC cases because the disease mostly affects older people. Thus, implementing automated reminders for people who are eligible, starting at 45 years, could also increase the uptake of screening.^20^

Furthermore, healthcare professionals are very important in the prevention of CRC through increasing awareness. It has been shown that advice from physicians is important for adherence to screening programs.^14^ A cross-sectional study found that people were likely to undergo screening when they had been directly advised by their doctor.^23^ Thus, empowering primary care physicians to discuss CRC screening options with their patients could contribute to increased participation. Moreover, it is important to address socio-cultural barriers such as fear, embarrassment, and incorrect beliefs about CRC and screening. Cultural sensitivity may be considered in interventions through social media, television campaigns, and community outreach programs in order to make screening practices more acceptable to the public.

Public health programs should enhance CRC awareness through targeted education campaigns, physician-led screening recommendations, and improved accessibility to screening services, particularly in peripheral areas. Studies in countries neighboring KSA, including UAE, Jordan, and Türkiye, have shown that public knowledge of CRC remains low because people do not understand the risk factors and physician do not adequately recommend screening tests.^24^, ^25^, ^26^ Therefore, healthcare organizations need to establish policies that connect knowledge acquisition with actual participation in screening.^24^, ^25^, ^26^

In the present study, we obtained some important insights into the awareness of CRC and screening behaviors specifically regarding targeted public health interventions in the Eastern Province of KSA. The strengths of this study include its region-specific focus, assessment of important demographic factors such as age, sex, education level, and family history, and the use of a validated survey instrument to enhance data reliability. Our findings showed that levels of knowledge about CRC and screening participation were low, thereby highlighting the need for more awareness campaigns and better access to screening.

However, the present study had some limitations. The study's focus on one region may hinder generalization of the results to the rest of KSA. In addition, the cross-sectional nature of the study allowed us to capture a snapshot in time but without establishing cause and effect. Moreover, data collected using questionnaires may be affected by recall and social desirability bias. Future longitudinal studies with a broader geographic coverage could help to improve our understanding of CRC awareness and screening practices in KSA. It should also be noted that the inclusion of some participants with medical backgrounds might have biased the awareness estimates.

## Conclusion

In the present study, we found low levels of CRC awareness and screening uptake among adults in the Eastern Province of KSA. Higher education, prior knowledge of CRC, and family history were significant predictors of good awareness, and older age and urban residence predicted screening uptake. To reduce the burden of CRC, future studies must evaluate the long-term impacts of awareness efforts and develop policy-driven interventions to bridge the gap between knowledge and participation in screening.

## Ethical approval

This study was conducted in accordance with the Declaration of Helsinki and approved by the Institutional Review Board at Imam Abdulrahman Bin Faisal University Dammam, KSA (IRB number: 2024-01-339) on 29/4/2024. Electronic informed consent was obtained from all participants voluntarily, with anonymity guaranteed.

## Authors contributions

Conceptualization, SAB; Data curation, BAA, MAA, LMB; Formal analysis, SAB, LMB, HAA; Investigation, BAA, LMB; Supervision, SAB; Writing – original draft, SAB, MAA; Writing – review & editing, SAA, HAA, OYA All authors read and agreed to the final version of the manuscript. All authors have critically reviewed and approved the final draft and are responsible for the content and similarity index of the manuscript.

## Source of funding

This study did not receive any specific grant from funding agencies in the public, commercial, or not-for-profit sectors.

## Conflict of interest

The authors have no conflicts of interest to declare.
